# Progress in Surface Modification of Titanium Implants by Hydrogel Coatings

**DOI:** 10.3390/gels9050423

**Published:** 2023-05-18

**Authors:** Huangqin Chen, Rui Feng, Tian Xia, Zhehan Wen, Qing Li, Xin Qiu, Bin Huang, Yuesheng Li

**Affiliations:** 1 Department of Stomatology, School of Stomatology and Ophthalmology, Xianning Medical College, Hubei University of Science and Technology, Xianning 437100, China; chenhuangqin79@163.com (H.C.);; 2 Hubei Key Laboratory of Radiation Chemistry and Functional Materials, Non-Power Nuclear Technology Collaborative Innovation Center, Hubei University of Science and Technology, Xianning 437100, China

**Keywords:** hydrogel coating, titanium alloy, biochemical modification, application

## Abstract

Although titanium and titanium alloys have become the preferred materials for various medical implants, surface modification technology still needs to be strengthened in order to adapt to the complex physiological environment of the human body. Compared with physical or chemical modification methods, biochemical modification, such as the introduction of functional hydrogel coating on implants, can fix biomolecules such as proteins, peptides, growth factors, polysaccharides, or nucleotides on the surface of the implants, so that they can directly participate in biological processes; regulate cell adhesion, proliferation, migration, and differentiation; and improve the biological activity on the surface of the implants. This review begins with a look at common substrate materials for hydrogel coatings on implant surfaces, including natural polymers such as collagen, gelatin, chitosan, and alginate, and synthetic materials such as polyvinyl alcohol, polyacrylamide, polyethylene glycol, and polyacrylic acid. Then, the common construction methods of hydrogel coating (electrochemical method, sol–gel method and layer-by-layer self-assembly method) are introduced. Finally, five aspects of the enhancement effect of hydrogel coating on the surface bioactivity of titanium and titanium alloy implants are described: osseointegration, angiogenesis, macrophage polarization, antibacterial effects, and drug delivery. In this paper, we also summarize the latest research progress and point out the future research direction. After searching, no previous relevant literature reporting this information was found.

## 1. Introduction

In the 1940s, some scholars implanted pure titanium (Ti) into the femur of mice without causing adverse reactions, which proved that Ti had good biocompatibility [1]. Later, more and more scholars began to apply pure Ti in dental implants, joint prostheses, and other clinical fields. However, in the process of application, it was found that the low hardness and poor wear resistance of Ti did not meet the requirements of the force parts of knee joint and hip joint, promoting research into and development of titanium alloys. Ti6Al4V is an α + β alloy with higher hardness, better wear resistance, and better workability compared with Ti (Table 1). However, aluminum (Al) and vanadium (V) in Ti6Al4V alloy are harmful metal elements, which have the risk of releasing after implantation into the human body. There is an urgent need to develop new medical titanium alloys with better biocompatibility. Representative materials are Ti–Nb–Zr and Ti–Nb–Zr–ME (Me (metal)) systems, especially Ti–Nb–Zr–Si (TNZS) alloy, which not only has better biocompatibility, but also has improved corrosion resistance, and can better match with human bone tissue [2].

At present, titanium and titanium alloys have become the preferred materials for medical metal products because of their lower density, higher specific strength, and better biocompatibility.

**Figure 1:**
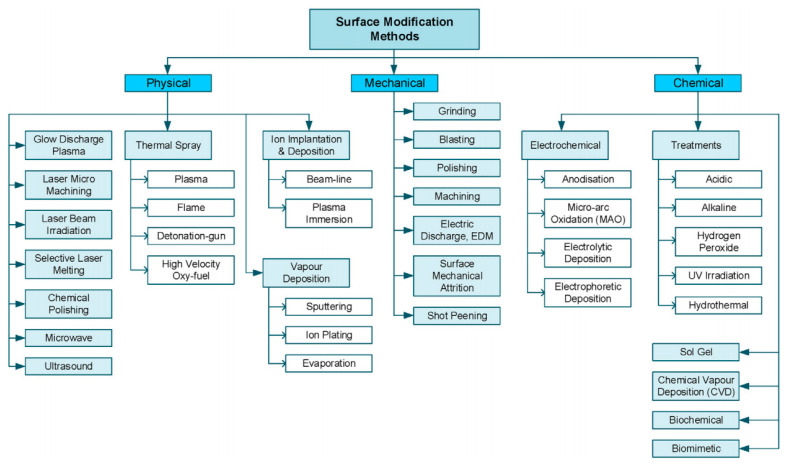
Surface modification methods of titanium and its alloys [3].

Although medical titanium and titanium alloys have outstanding properties, it is still necessary to strengthen the research on surface modification technology to match the complex physiological environment in human body (Figure 1) [3]. Now, construction of functional coatings on titanium and titanium alloys has attracted more and more attention. For example, titanium nitride [4], titanium aluminum nitride [5], and titanium dioxide [6] coatings significantly improve wear resistance and corrosion resistance of titanium and titanium alloys. The modification of Si–TiO_2_ nanotubes on the Ti substrate generates a nanostructured and hydrophilic surface, which can promote cell growth. Moreover, the existence of the TiO_2_ nanotubes and Si element improves the in vitro osteogenic differentiation of MC3T3-E1 cells and early bone-formation around the implanted screws [7]. Coating of silver (Ag), copper (Cu), zinc (Zn), and other antibacterial metal elements show excellent antibacterial properties [8,9]. Adding Cu with Ti-15Mo reduces the possibility of bacterial infection during biomedical implant surgeries [10].

Differently from physical or chemical modification methods such as micro-arc oxidation and sandblasting to prepare oxide film or rough surface on the surface of implants, biochemical modification of fixing specific proteins, peptides, growth factors, polysaccharides, nucleotides, and other biomolecules on the surface of implants can directly participate in biological processes and regulate cell adhesion, proliferation, migration, and differentiation [11,12]. Hydrogel is a type of hydrophilic three-dimensional network structure formed by natural or artificial synthetic polymer materials through the gelation process of sol, which is widely used in many fields such as tissue engineering, drug delivery, and biosensors. The functional hydrogel coatings on titanium implants can effectively coordinate the advantages of hydrogel (lubricity, biocompatibility, and controlled release) with those of implants (stiffness, strength, and toughness) [13], and change the electrochemical behavior of titanium implants and enhance corrosion resistance [14].

In this review, we will first introduce the classification of hydrogel coatings on the surface of titanium implants, including natural hydrogels and synthetic hydrogels, according to the composition of the hydrogel matrix. We will then introduce the common binding methods of hydrogel coatings and titanium implants, such as the electrochemical precipitation method, the sol–gel method, and the layer-by-layer self-assembly method. Subsequently, the improvement of titanium implants by hydrogel coating on osseointegration, angiogenesis, macrophage polarization, antibacterial, and drug delivery are summarized in detail. Finally, the possible problems and future development direction of hydrogel coatings are presented in order to provide reference for scientific research workers in related fields. After searching, no previous research reporting of this information was found.

## 2. Classification of Hydrogel Coatings

According to the main components of the hydrogel matrix, hydrogel coatings can be divided into natural hydrogel coatings and synthetic hydrogel coatings (Table 2).

### 2.1. Natural Hydrogel Coating

Natural hydrogel is composed of natural biological materials which are highly similar to the extracellular matrix. It is considered as good biomimetic material in tissue engineering because of its complete bioactivity in promoting cell adhesion, proliferation, differentiation, and biodegradation. Common natural biomaterials are collagen and gelatin from animal protein, hyaluronic acid from animal epithelium and connective tissue, chitosan from shells of crustaceans, and alginate from the cytoplasm and cell wall.

#### 2.1.1. Collagen-Based Hydrogel Coating

**Figure 2:**
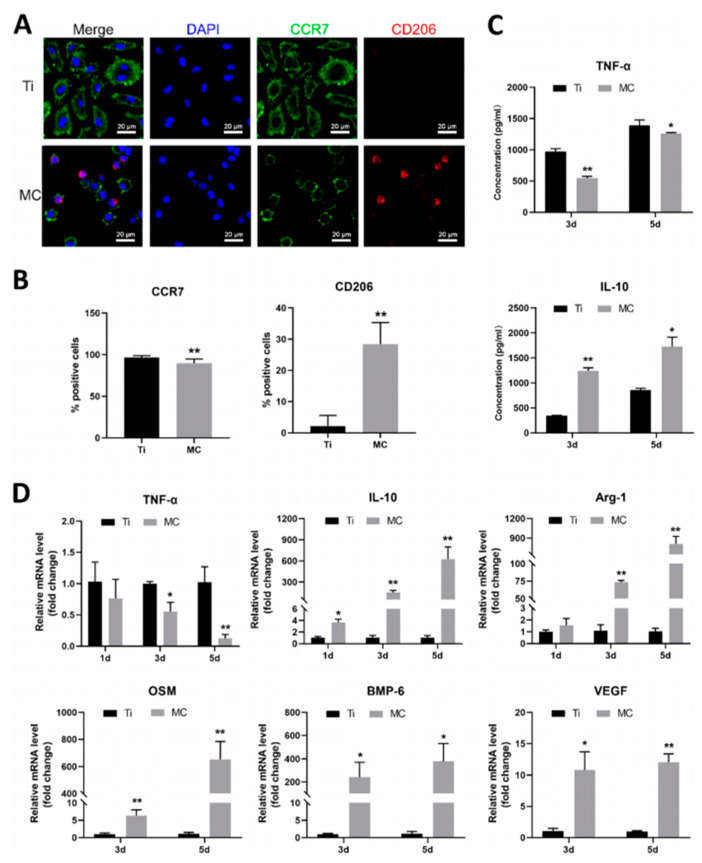
Functional evaluation for BMDM polarization through surface biomarkers (**A**,**B**), inflammatory factor secreting pattern (**C**), and related gene expression levels (**D**). CCR7 and TNF-α served as the M1-polarized markers; CD206, IL-10, and Arg-1 served as the M2-polarized markers; OSM, BMP-6, and VEGF served as the pro-regeneration biomarker. * *p* < 0.05; ** *p* < 0.01 [43].

Collagen is the main component of the extracellular matrix in mammals, and is mainly distributed in the cornea, cartilage, bone, blood vessels, viscera, intervertebral discs, and dentin, and plays an important role in supporting and protecting the body and organs. Collagen has the advantages of non-cytotoxicity, good biocompatibility, easy absorption, small immune response, low antigenicity, etc. Coating titanium alloys with collagen promotes adhesion, proliferation, and differentiation of born-forming cells [30,31,32,33] as well as fibroblasts [34,35]. In comparison with uncoated commercially pure titanium, collagen coating significantly improves bone mineralization and maturation [36]. More rapid osteointegration will be achieved if the coating is combined with vitamins [37], phospholipid [38], or hydroxyapatite (HA) [39,40,41]. Besides osteogenesis, collagen coating can support the timely conversion of macrophages from the pro-inflammatory M1 to the pro-healing M2 phenotype, inhibiting inflammatory reaction and generating a beneficial osteoimmune microenvironment [42,43] (Figure 2). Simultaneously, collagen coating prominently facilitates angiogenesis of endothelial cells [42] and strengthens local blood supply restoration through sustained release of vascular endothelial growth factor (VEGF) [44].

#### 2.1.2. Gelatin-Based Hydrogel Coating

**Figure 3:**
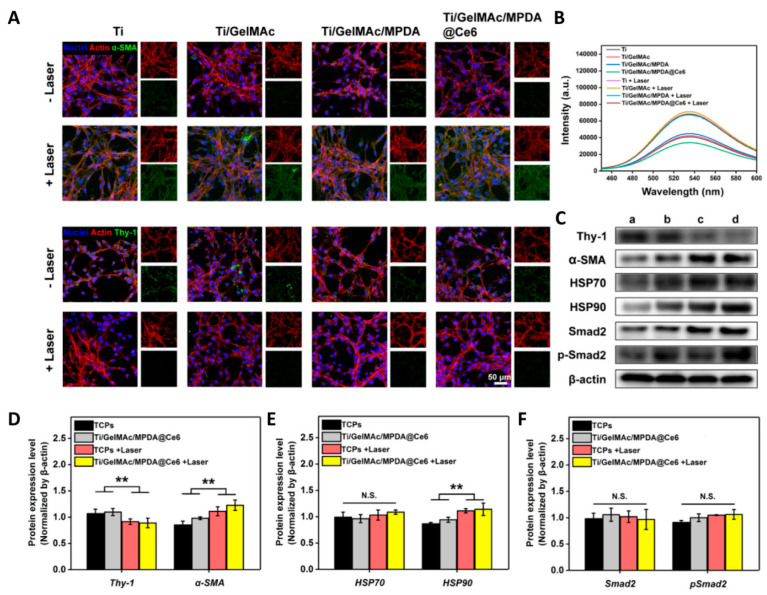
Fibroblast activation, or fibroblast-myofibroblast transition, of NIH/3T3 cells after irradiation [50]. (**A**) Representative fluorescence images of NIH/3T3 cells cultured with different samples for 2 days. The expression of α-SMA and Thy-1 was stained as green. (**B**) Cellular ATP level reflected by luminescence intensity after different treatments. (**C**) Western blotting detecting the expression of Thy-1, α-SMA, HSP70, HSP90, Smad-2, and p-Smad-2 after different treatments. a: TCPs; b: Ti/GelMAc/MPDA@Ce6; c: TCPs + Laser; d: Ti/GelMAc/MPDA@Ce6 + Laser. (**D**–**F**) Quantitative analysis according to Western blotting results. (n = 6, ** *p* < 0.01, N.S.: no significance). (For interpretation of the references to color in this figure legend, the reader is referred to the Web version of this article.)

Gelatin is a product of collagen hydrolysis but retains arginine–glycine–aspartic (RGD) cell adhesion peptide and protease degradation sites of collagen with lower immunogenicity. Gelatin-based hydrogel coatings enhance the integration between implant and tissue [45], and promote biological activity by loading various growth factors. For example, gelatin coating loaded with VEGF/bone morphogenetic protein 2 (BMP-2) shell-core microspheres promoted osteogenic differentiation and osseointegration effectively in 3D-printed porous titanium alloy [46]. In 2000, Van den Bulcke et al. introduced the methacryl group into modified gelatin for the first time to prepare methacryl amide gelatin, which gave the gelatin the property of photo-cross-linking under the photoinitiator and light [47]. When loaded with a short cationic antimicrobial peptide and synthetic silicate nanoparticles, the photo-cross-linked gelatin-based hydrogel coating demonstrated excellent antimicrobial activity and enhanced osteogenesis [48]. The addition of ginger inhibited the growth of *S. mutans* and *P. gingivalis* [49]. Methylacrylamide gelatin combined with photosensitizer and photocatalyst offers direct fibroblast activation [50] (Figure 3) and multi-mode photothermal and photodynamic antibacterial effects [51]. Furthermore, the allylated gelatin co-encapsulated human umbilical vein endothelial cells (HUVECs) and human mesenchymal stromal cells (hMSCs) support and achieve concurrent vasculogenic and osteogenic performance [52].

#### 2.1.3. Chitosan-Based Hydrogel Coating

**Figure 4:**
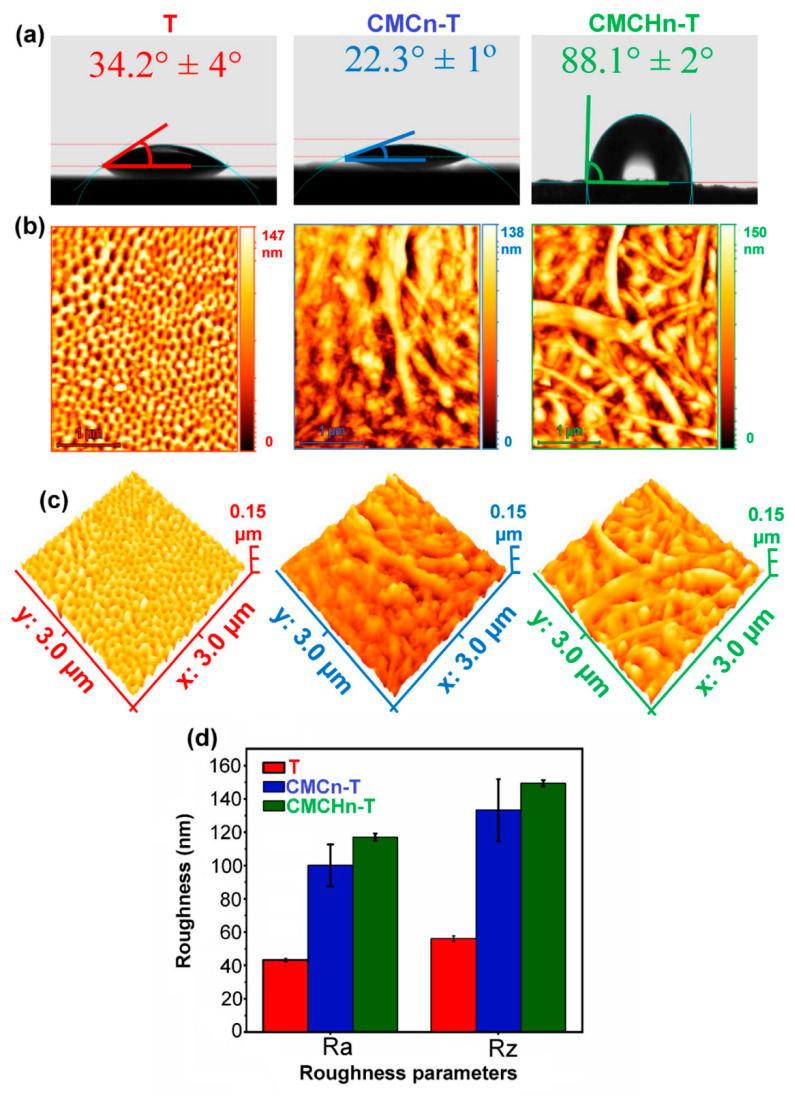
Wettability and topography of the implant’s surface [77]. (**a**) Contact angle values and water droplet images, (**b**,**c**) 2D and 3D AFM images, and (**d**) average maximum height of the profile (Rz) and the arithmetic mean roughness (Ra) of the synthesized samples (*p* < 0.05).

Chitosan, the deacetylated chitin, is the only natural alkaline polysaccharide with charge. Due to their pH, ionic strength, and temperature sensitivity, chitosan-based hydrogels have good application prospects in the fields of targeting, sustained drug release, tissue engineering, and medical dressings [53]. Chitosan-based hydrogel coatings increase the antibacterial ability of the implant by loading antibacterial agents [54,55,56], or metal ions (Ag, Cu) [57,58,59,60]. Coatings give the implant the photocatalytic antibacterial effect by modifying or loading novel semiconductor materials, such as graphene [61], molybdenum disulfide [62], black phosphorus [63], and molybdenum diselenide [64]. They also promote osteogenesis through loading drugs, (for example, pitavastatin [65] and quercetin [66]), active substances (insulin growth factor binding protein-3 [67] and BMP-2 [68]) and inorganic matter (HA [69,70] and bioactive glass [71]). In addition, chitosan combines with polyanions such as gelatin [72,73], hyaluronic acid [74], and sodium alginate [75,76] to form a polyelectrolyte complex, promoting the surface functionalization of titanium. Modified carboxymethyl chitosan nanofibers, as a novel implant coating on titania nanotube arrays, inhibit bacterial colony formation and increase osteoblast cell survival [77] (Figure 4). Similarly, carboxymethyl chitosan loaded with silver nanoparticles enhances the antibacterial properties of the titanium alloy [78].

#### 2.1.4. Alginate-Based Hydrogel Coating

Alginate is a type of linear hydrophilic polysaccharide existing in brown algae. It forms hydrogels by ionic cross-linking with Ca^2+^ and other polyvalent inorganic cations. There are a large number of –OH and –COOH groups on the alginate skeleton, which can be modified by chemical or physical methods to achieve controlled release of cells or bioactive molecules in response to temperature, pH, and light [79]. Composite coating formed by alginate crosslinking with collagen enhances the cell adhesion of titanium implants [80,81]. Alginate and chitosan coating improves the biomineralization, the antibacterial activity, and corrosion resistance [75,82]. The addition of Ag further promotes the antibacterial ability and reduces the bacterial adhesion [83,84]. Alginate-based hydrogel coatings also provide sustained antibacterial activity by loading various antibacterial agents such as gentamicin [68], vancomycin [85], and chlorchloridine [86], and improving in vitro osteogenic differentiation as well as bone integration by loading BMP2 [87] and RGD [88].

### 2.2. Synthetic Hydrogel Coatings

The synthetic hydrogels have great application potential due to the wide source of raw materials, simple synthesis method, and controllable composition and structure. Synthetic hydrogels can be divided into the following functional groups: non-ionic hydrogels including polyvinyl alcohol (PVA), polyacrylamide (PAM), poly N-isopropylacrylamide (PNIPAm), polyethylene glycol (PEG), poly (lacto-glycolic acid) (PLGA), etc., and ionic hydrogels such as polyacrylic acid (PAA).

PVA hydrogels with porous titanium bases are being developed to repair or replace articular cartilage due to their high mechanical strength [89]. The Ti–hydrogel artificial cartilage material constructed with polydopamine (PDA), PVA, HA, or PAA as raw materials is an ideal high-strength and low-friction biomimetic cartilage material [90]. The novel “soft (PVA hydrogel layer)–hard (porous Ti6Al4V alloy substrate)” structure improves the surface wettability and tribological properties of Ti6Al4V alloy [91].

PAM hydrogel is an injectable soft-tissue-filling material. PAM-based hydrogels in combination with titanium-oxide nanotubes are also widely used as potential candidates for cartilage replacement [92]. PAM/PVA hydrogel on Ti6Al4V alloy configuration combines the good load-bearing capacity of the rigid substrate and the excellent lubrication of the hydrogel layer [93]. The cross-linked network porous structure of hydrogel is the main factor accounting for the low dynamic friction [94].

PNIPAm is a thermo-responsive polymer with lower critical solution temperature (LCST) around 32 °C. When the temperature is above the LCST, the polymer chains become hydrophobic and collapse, resulting in dense crosslinking networks in which the loaded molecules are more likely to be trapped, thus leading to slow release [95].

PEG is obtained by glycol dehydration polycondensation. The functional-group-hydroxyl at the end of the molecular chain is prone to chemical reactions and chemical modifications [96]. PLGA–PEG–PLGA hydrogels, polymerization of PLGA with PEG, are suitable for drug loading in vitro and sustained drug release in vivo, owing to the thermo-sensitive properties [97].

PAA hydrogels are three-dimensional macromolecules containing a large number of carboxyl groups that cannot move freely. When the pH value of the solution is different, it presents different degrees of shrinkage or swelling state. It can be used to prepare a simple and low-cost hydrogel-based bone adhesive to improve the osseointegration and anti-infection ability of the bone-implant interface [98]. 

## 3. Binding Method of Hydrogel Coating and Titanium Implant (Preparation Method of Hydrogel Coating)

Titanium implants have some disadvantages and one of the effective strategies is to prepare multifunctional hydrogel coatings on the surface. The most commonly used preparation method is sol–gel method.

### 3.1. Electrochemical Methods

**Figure 5:**
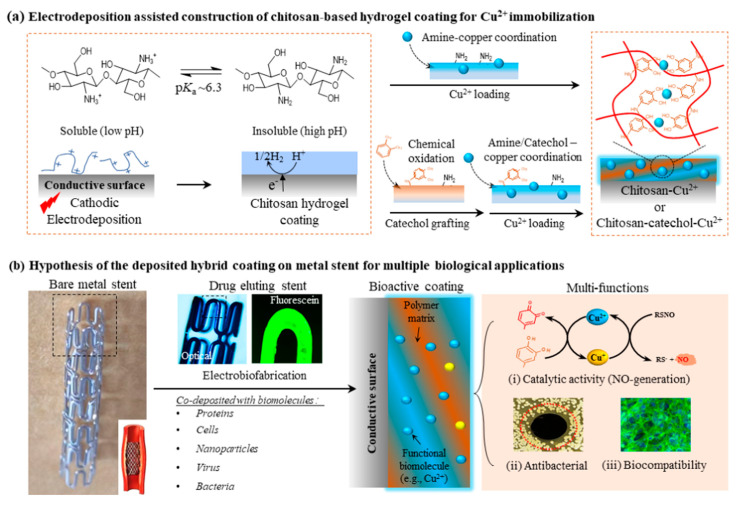
Electrochemical construction of a catechol-grafted chitosan film for Cu^2+^ incorporation [101].

The electrochemical methods for preparing hydrogel coatings mainly include electrochemical deposition and electrophoretic deposition. Electrochemical deposition refers to the process of forming coatings on the surface of metals or alloys in aqueous or non-aqueous solutions of inorganic salts and bio-active factors, which is a promising technique for surface modification of implants with various shapes, especially deformed structures [99,100]. The chitosan hydrogel coating, which is a versatile platform for Cu immobilization and precisely controlled synthesis via electrochemical deposition, has in vitro cell biocompatibility and catalyzed nitric-oxide-generation activity [101] (Figure 5). Electrophoretic deposition refers to the phenomenon of powder particles deposited from the suspension on the electrodes with opposite charges and certain shapes, relying on the action of the direct current [102]. The lanthanum- and silicate-substituted composite coating on a titanium implant achieved by the electrophoretic deposition method exhibited strong osteogenic ability [103]. Moreover, UV irradiation can be used as a crosslinking activator to deposit gentamicin-loaded agarose hydrogels, controlling the release of the loaded antibacterial agents while improving cell integration [104]. 

### 3.2. Sol–Gel Method

**Figure 6:**
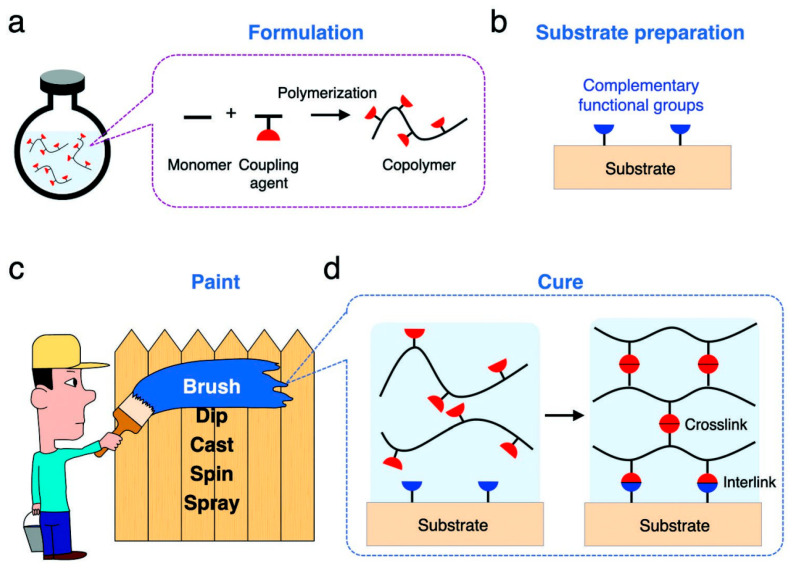
Principle of hydrogel coating [105]. (**a**) Formulation: monomer units and coupling agents copolymerize into polymer chains, but do not crosslink into a network, resulting in an aqueous solution. The solution may also contain other compounds for various functions but are not drawn here. (**b**) Substrate preparation: functional groups complementary to the coupling agents are imparted onto the surface of a substrate. (**c**) Paint: The aqueous solution is painted on the substrate by various operations. (**d**) Cure: The coupling agents react with each other to crosslink the polymer chains into network and react with the complementary functional groups to interlink the polymer network to the substrate.

Hydrogel coatings are mostly prepared by the sol–gel method. That is, monomers and coupling agents, as well as substances with different functions (initiators, loaded drugs, etc.), are dissolved in water to generate a free-radical polymerization reaction to form uncross-linked polymer chains. Then, the formulated aqueous solution is coated on the prepared matrix, and the polymer chain is cross-linked into a polymer network by the coupling agent. Finally, the polymer network is connected to the matrix by reacting with complementary functional groups on the matrix surface (Figure 6) [105].

Common methods of applying aqueous solution to the substrate are spraying [106], spin [26,107], and impregnated lift [52,108]. In order to enhance integration between implant material and hydrogel, a PDA layer was introduced onto the surface of the titanium alloy. Through chemical crosslinking between PDA and gelatin [45,51] or HRP/H_2_O_2_ catalysis [78], the hydrogel precursor could simply form a firm gel layer on the titanium alloy plate. Methylacryylated gelatin (GelMA) is a photo-cross-linked gelatin derivative. The photoinitiator [109] or catechol motifs [50,110] stabilize the GelMA hydrogel system and make the coating tightly adhere to titanium substrates after 365 nm UV exposure. The sol–gel method caused by ionizing radiation is a safe, simple operation with no polluting effects. Unfortunately, this method is rarely used in the preparation of hydrogel coatings on titanium and titanium alloys.

### 3.3. Layer-by-Layer Self-Assembly 

**Figure 7:**
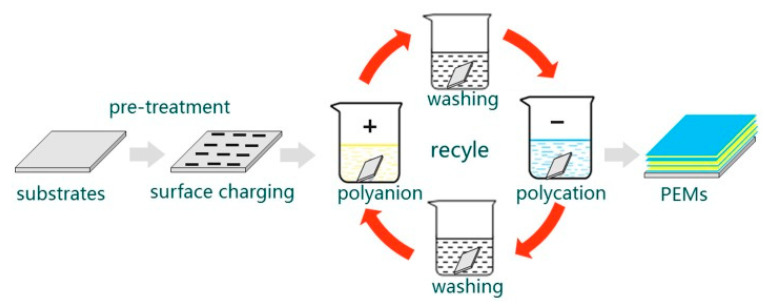
Layer-by-layer electrostatic self-assembly [112].

Layer-by-layer self-assembly (LBL) is a popular surface modification method that uses electrostatic adsorption to self-assemble layers of materials with opposite charges into multilayer structures [111] (Figure 7). The assembly process is simple and gentle, and can maintain the biological activity of cytokines and achieve sustained release drug delivery [112]. However, it is necessary to pretreat the Ti surface with microarc oxidation, electrochemical deposition technology [113], etc., to firmly immobilize the multilayers. As one of the silyl reagents, 3-aminopropyl triethoxysilane is often used to aminofunctionalize titanium substrates, promoting covalent coupling to form precursor layers and facilitating the construction of future multilayer coatings [114]. The titanium alloy surfaces can also conjugate with dopamine as the base layer, which enables the deposition of gelatin molecules of hydrogel precursor [115]. PDA is a common mussel-inspired anchoring polymer and exhibits powerful reactivity to various bioactive molecules containing carboxyl groups, amino groups, and thiol groups. A multilayer type-I-collagen decorated nanoporous network was successfully developed on alkali-treated titanium surfaces via PDA coating and LBL [42]. The phase-transited lysozyme provides a new approach to achieving a high binding force that is superior to dopamine, and which forms an amyloid-like microfiber net that tightly adheres to Ti surfaces according to the transition process of lysozyme based on the β-sheet of lysozyme microfibers [116,117]. In addition, tannic acid is a low-cost plant polyphenol, which can bind materials tightly via hydrogen bonds, Michael addition reactions, Schiff base reactions, etc., due to the composition of a glucose core and a hydroxyl-rich phenolic shell, show great potential in LBL [118]. 

## 4. Characterization Methods of Surface Modification

Characterization methods for hydrogel coatings include nuclear magnetic resonance (NMR), Fourier transform infrared spectroscopy (FTIR), X-ray diffraction (XRD), and scanning electron microscopy (SEM). The position of the resonance signal on the NMR spectrum reflects the local structure of the sample molecules, such as functional groups. FTIR is the absorption spectrum generated by the absorption of specific wavelengths of infrared light during the vibrational energy level transition of bond-forming atoms in compound molecules, and is mainly used for structural analysis, qualitative identification, and quantitative analysis. XRD is the crystal structure analysis of hydrogel precursor polymer, such as fibroin protein, collagen, and other natural macromolecules containing crystal structure, or loaded nanoparticles such as biological glass and HA. Moreover, SEM and 3D optical profilometer are used to detect the morphological changes and thickness of hydrogel coating, respectively.

## 5. Application of the Hydrogel Coating

### 5.1. Osseointegration

Osseointegration is the direct contact between the implant and the bone tissue under the optical microscope, without fibrous connective tissue. Good osseointegration is a key factor in the long-term success of implants. Physical and chemical modification methods, such as changing surface properties [119] and loading inorganic substances, mainly indirectly affect cell behavior, with limitations in improving osteogenic activity [120,121]. Biochemical modifications caused by biomolecules [122,123,124,125,126,127] immobilized on the surface of titanium implants directly participate in biological processes and are more effective in inducing bone-formation, especially in poor bone conditions [128]. 

**Figure 8:**
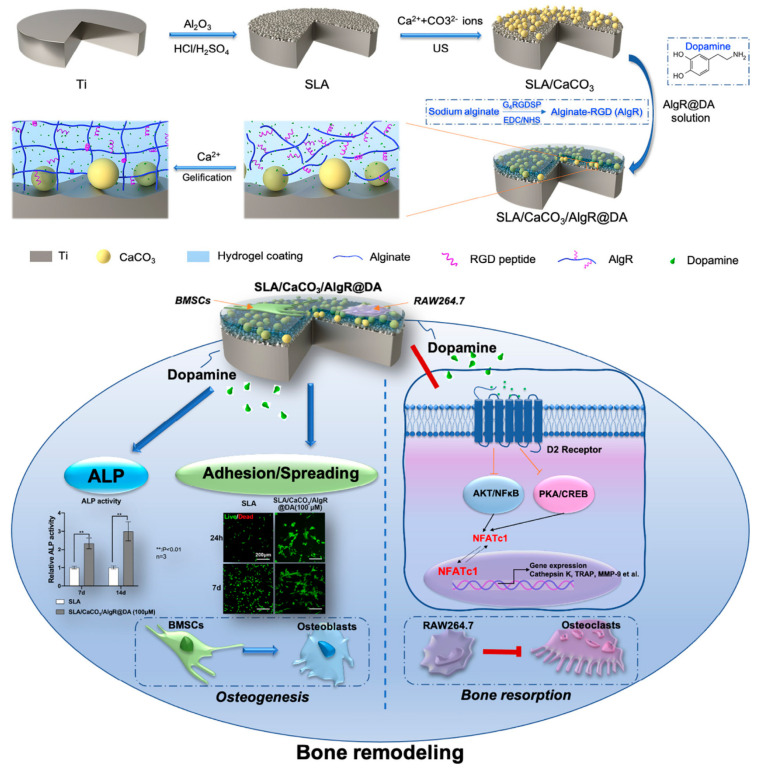
Hydrogel coatings on titanium bidirectionally regulate osteoclastic and osteogenic response behaviors [88].

Hydrogel is a three-dimensional network cross-linked structure, which can not only simulate the extracellular matrix environment and develop bio-mimetic implants to design and repair bone defects [129], but also serve as drug carrier to carry and slowly release various active substances to promote bone-formation. Poloxamer-407 hydrogel loaded with simvastatin induces endogenous osteogenic growth factors and promotes bone ingrowth [130]. Pluronic F-127 hydrogel controls the release of 1α,25-Dihydroxyvitamin D3 as a bio-cap [131]. A non-toxic click hydrogel that rapidly polymerizes in situ provides localized controlled delivery of osteoprotective factor Semaphorin 3A [132]. Hydrogel containing BMP-2 facilitates dimensionally stable bone regeneration [133]. Dopamine-loaded RGD coatings on a vaterite-modified titanium surface successfully provided a solution to bone remodeling imbalance in osteoporosis by promoting osteoblasts and inhibiting osteoclasts at different concentrations [88] (Figure 8). Titanium implants loaded with human bone marrow mesenchymal stem cells (hBMSCs) show superior tissue ingrowth, and the synergic action of the bioactive hydrogel and hBMSCs increases both the bone deposition and integration [134]. In addition to these active ingredients, some inorganic substances such as a tri-calcium phosphate- [135], HA- [136], and silica-nanoparticle-loaded [137] hybrid hydrogels also improve the osteogenic ability of titanium implants, especially in combination with BMP-2 [138] or osteoblasts [139].

### 5.2. Angiogenesis

**Figure 9:**
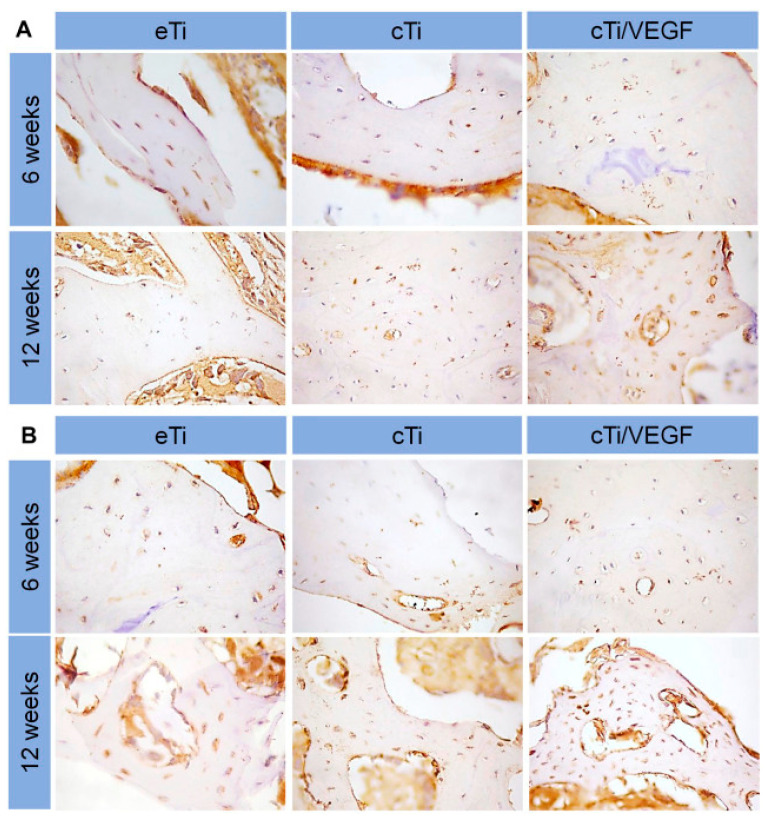
Sustained release of VEGF promotes COL I (**A**) and CD 31 (**B**) expression in bone-surrounding scaffolds 6 and 12 weeks after implantation (n = 3) [44].

Adequate blood supply plays an indispensable role in promoting bone regeneration, and angiogenesis promotion has become one of the key factors for the success of titanium implants. Hydrogels can act as carriers for drugs, growth factors, and cells, to promote angiogenesis around titanium implants. The combination of simvastatin-loaded hydrogel coating with porous titanium alloy significantly improved the formation of new blood vessels around rabbit tibial implants, providing an effective strategy for bone integration and bone growth [140]. The heat-sensitive collagen hydrogel/porous titanium alloy scaffold system equipped with VEGF, increased vascular permeability, promoted proliferation and induction of HUVECs, and aided in angiogenic-mediated bone regeneration [44] (Figure 9). The composite scaffold loaded with VEGF and BMP continuously provided angiogenic and osteogenic growth factors at the site of osseous defect, thus exhibiting higher bone integration capacity and new bone amount [46,141]. Combining cell-laden hydrogels with porous titanium alloys develops a vascularized bone implant. Co-encapsulating hMSCs with HUVECs [52] or endothelial progenitor cells (EPCs) [142], support HUVEC- and EPC-spreading and vascular-like network formation, along with osteogenesis of hMSCs.

### 5.3. Macrophage Polarization

**Figure 10:**
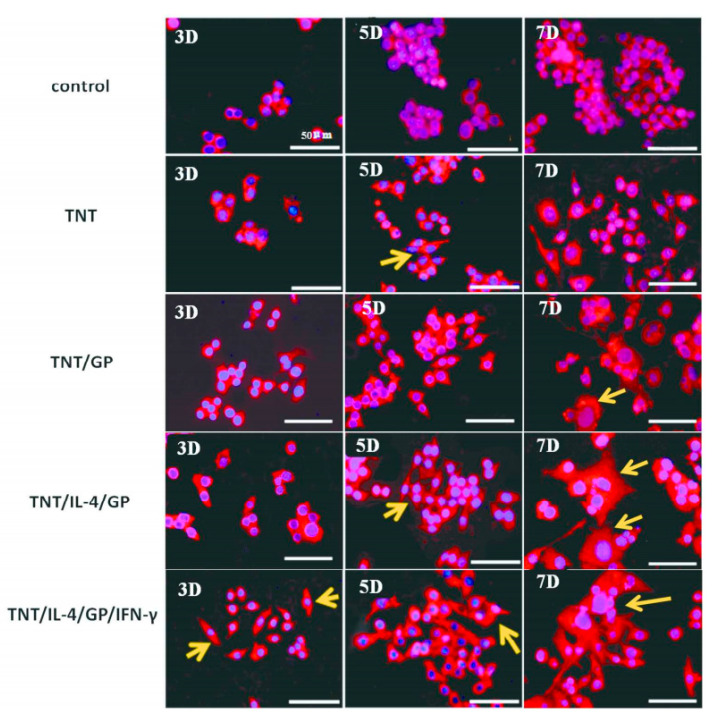
Fluorescence images of macrophage morphologies on dual-inflammatory cytokine (interferon-γ and IL-4)-coated TiO_2_ nanotube surfaces [144]. Activated macrophages are indicated by yellow arrows.

Macrophage polarization is a reversible and modified dynamic process involving in the occurrence, development and outcome of many immune inflammatory diseases, including peri-implantitis. The introduction of hydrogel for “reprogramming” of the macrophage state is a novel strategy to induce resolution of inflammation [143]. Interleukin-4 (IL-4) is a common inflammatory factor, which can regulate the antigen-presenting ability of macrophages, inhibit the secretion of inflammatory factors such as IL-1 β and TNF- α, and promote the differentiation of macrophages into profibrotic macrophages to secrete TGF-β. IL-4-loading of a hydrogel system on titanium modulated pro-inflammatory reactions [110]. Hydrogels containing interferon-γ and IL-4 were able to modulate the transformation with a stronger effect than those containing only IL-4 [144] (Figure 10). Combination of IL-4 and cell adhesive motif (RGD) onto the Ti substrate synergistically generated a more favorable early-stage osteo-immune environment with superior osteogenic properties [145]. Dexamethasone, as a glucocorticoid, can also regulate macrophage polarization and plays an important role in the regression of inflammation. The novel DNA hydrogel on the titanium surface, as the platform for dexamethasone delivery, extends the half- life of the release profile [146]. Reactive oxygen species (ROS) produced by macrophages regulate a variety of physiological functions including endothelial cells growth, migration, and mesenchymal stem cells activation. Removing excessive ROS by a two-component hydrogel coating containing borate ester bond and thymosin β4 favors M1 to M2 phenotype switch of macrophages and inflammatory response regulation [147].

### 5.4. Antibacterial

Bacterial biofilm formation can cause implant infection and osseointegration loss, resulting in loosening and dropping. Hydrogels with good biocompatibility and drug loading capability can slowly release various antibacterial components to prevent initial bacterial adhesion [13]. Designing and constructing a hydrogel drug-controlled release system by loading with antibacterial drugs such as gentamicin [104,108,148] or vancomycin [98,149,150] on a titanium surface is a frequently used strategy. Antibacterial peptides have garnered more attention as alternative antibacterial agents of implant coating due to their unique antibacterial mechanism [151,152]. Bacteriophage-loaded hydrogels also showed excellent antimicrobial activity in inhibiting attachment and colonization of multidrug-resistant *E. faecalis* surrounding and within femoral tissues [153]. Metal antibacterial agents are introduced into the implant hydrogel coating because of their broad-spectrum antibacterial properties and no drug resistance. Among them, silver ion is most commonly used [26,78,154,155]. Metal oxide antimicrobial agents such as zinc oxide [109] and calcium oxide [110] also show significant antibacterial ability in the coating, although Zn ion has renal absorption toxicity. Photodynamic therapy is a promising modality in antibacterial material design. The introduction of photosensitizer Chlorin e6 with laser-triggered ROS generation property exhibited a remarkable and rapid antibacterial activity when the laser power was 1 W cm^−2^ [50]. Coatings with semiconductor photocatalytic materials, such as bismuth [51] and red phosphorus [156], can produce ROS, kill bacteria and eradicate biofilm under light, which might provide a novel multimodal antibacterial and anti-biofilm treatment for infection. 

### 5.5. Drug Delivery

Hydrogels have been widely used in various fields of medicine as vehicles to control the continuous release of drugs [157]. Loading cefuroxime, tetracycline, amoxicillin, or acetylsalicylic acid through hydrogel coating can improve the anti-infection effect of the implant [158]. Loading bone-metabolism-related drugs, proteins, peptides, and growth factors has demonstrated better osseointegration, especially in challenged degenerative conditions, such as osteoporosis, osteoarthritis, and osteogenesis imperfecta [159]. Similarly, hydrogels can load with cytokines to promote macrophage polarization [160] and angiogenesis [141], which have great potential for application in bone-tissue regeneration and repair.

## 6. Conclusions and Future Protects

In recent decades, metallic materials have been widely used in the field of biomaterials for their good mechanical properties and biocompatibility. Among them, the application prospects of biomedical titanium alloy are particularly remarkable. However, some disadvantages of titanium alloys limit their further application. Therefore, scientists have been working to explore improvements in the properties of titanium alloys. Hydrogel coatings can serve as ideal carriers to introduce drugs, peptides, metal ions, growth factors, and cells to effectively bio-modify titanium alloys. In this study, we have reviewed the popular matrix of hydrogel coatings, especially the natural materials such as collagen, gelatin, chitosan, and alginate. The usual modification methods are the electrochemical method, the sol–gel method, and layer-by-layer self-assembly. Hydrogel coatings significantly improve the properties of the titanium implant in osseointegration, angiogenesis, macrophage polarization, antibacterial effects, and drug delivery.

Although the improvement of titanium alloy caused by hydrogel coating is obvious, there are still some problems worth noting: (1) The mechanical properties of hydrogel coating are poor, and whether some inorganic fillers can be added to promote bone integration and mechanical properties needs further research. (2) The strong bond between the hydrogel coating and titanium alloy needs to be further strengthened. (3) Some semiconductor materials have excellent photocatalytic properties, and the introduction of semiconductor materials in hydrogel coatings is promising for photodynamic therapy. Therefore, future research should focus on these aspects to further improve the properties of hydrogel coating on titanium alloy.

## Figures and Tables

**Table 1:** Development of medical titanium and titanium alloys.

Classification	Time	Representative Material	Advantage	Disadvantage
α	1960s	Ti	Good biocompatibility	Low strength, poor wear resistance
α + β	1970s	Ti6Al4V	Higher hardness, better wear resistance, lower elastic modulus, better mechanical compatibility	Biological toxicity of metal ions Al and V
	1980s	Ti6Al7Nb Ti5Al2.5Fe	Better biocompatibility	Easy corrosion, biological toxicity of Al metal ions
β	1990s	Ti13Nb13Zr Ti12Mo6Zr2Fe Ti15Mo	The low modulus of elasticity is close to that of human bones, non-biological toxicity of metal ions	Biological activity, abrasion resistance, and corrosion resistance still need to be improved

**Table 2:** Classification of hydrogel coatings.

Classification	Representative Material	Advantage	Reference
Natural hydrogel coatings	Collagen-based	Improve the attachment of the peri-implant soft tissue to titanium at early stages	[15]
		Enhance tissue vascularization and reduce inflammatory response	[16]
		Improve gingival connective tissue response to titanium implants	[17]
	Gelatin-based	Improve surface bio-activity	[18]
		Load with antibacterial agent curcumin	[19]
	Chitosan-based	Enhance the antibacterial activity and osteoinductive properties	[20]
		Develop a close bony apposition or the osseointegration of dental/craniofacial and orthopedic implants	[21]
		Provide a self-protective surface that prevents bacterial colonisation and implant-associated infections	[22]
		Great potential in implant anticorrosion	[23]
	Alginate-based	Improve the antibacterial effect and induce mineralization of dental implants	[24]
		Successively functionalize titanium surface	[25]
Synthetic hydrogels coatings	Polyvinyl alcohol	Improve the calcium silicate coating-to-substrate adhesion.	[26]
	Polyacrylamide	Antimicrobial-loaded hydrogel coatings	[27]
	Polyethylene glycol	Lower albumin adsorption and presented a decreased fibroblast, *Streptococcus sanguinis* and *Lactobacillus salivarius* adhesion.	[28]
	Poly (lacto-glycolic acid)	Drug release	[29]
	Polyacrylic acid	Acts as both an effective bioactive surface and an effective anti-corrosion barrier	[14]

## Data Availability

Not applicable.
